# Serotyping and molecular profiles of virulence‐associated genes among *Klebsiella pneumoniae* isolates from teaching hospitals of Ardabil, Iran: A cross‐sectional study

**DOI:** 10.1002/hsr2.1557

**Published:** 2023-09-12

**Authors:** Neda Same Maleki, Forough Babazadeh, Mohsen Arzanlou, Roghayeh Teimourpour, Hadi Peeri Dogaheh

**Affiliations:** ^1^ Department of Microbiology, School of Medicine Ardabil University of Medical Sciences Ardabil Iran

**Keywords:** capsular serotype, Iran, *Klebsiella pneumoniae*, serotyping, virulence genes

## Abstract

**Background and Aims:**

*Klebsiella pneumoniae* is a Gram‐negative bacterium that colonized various organs. This bacterium is associated with different community‐acquired and hospital‐acquired infections. The present study aims to assess the capsular serotypes and frequency of virulence‐associated genes in *K. pneumoniae* isolates from teaching hospitals in Ardabil, Iran.

**Methods:**

From October 1, 2019, to November 31, 2021, different clinical samples were collected and *K. pneumoniae* isolates were diagnosed using conventional biochemical tests. The final identification of *K. pneumoniae* was performed through the polymerase chain reaction (PCR) method using a specific primer targeting the *khe* gene. The PCR method was employed to confirm the presence of virulence‐associated genes and aerobactin, and the main capsular serotypes based on the specific primers.

**Results:**

Of all 100 *K. pneumoniae* isolates, 4% and 2% were typeable with K5 and K2 primers, respectively. In addition, *entB* (94%), *fimH* (91%), and *wcaG* (87%) had the highest frequency among the virulence‐associated genes. 24% of *K. pneumoniae* isolates harbored the *entB‐wcaG‐fimH* genes simultaneously. Moreover, 50% of capsular serotype 5 harbored the *ybts‐mrkD‐entB‐wcaG‐fimH* genes simultaneously.

**Conclusion:**

The findings revealed that 6% of all *K. pneumoniae* isolates were typeable, distributed in the two serotypes K5 and K2. Most *K. pneumoniae* isolates were positive for multiple types of virulence genes. Identifying bacterial virulence genes aids in molecular detection, assay development, and therapeutic pathways.

## 1 INTRODUCTION

*Klebsiella pneumoniae* is an opportunistic Gram‐negative bacterium belonging to the *Enterobacteriaceae* family that can normally colonize several organs such as the oropharynx, skin, and gastrointestinal tract.^1^, ^2^ This bacterium is associated with different community‐acquired and hospital‐acquired infections. The range of infections caused by *K. pneumoniae* varies from urinary tract infections, soft‐tissue infections, pyogenic liver abscess, pneumonia, endocarditis, and meningitis to severe bacteremia, especially in patients with chronic disease or immunodeficiency disorders.^3^, ^4^, ^5^

In 2017, the US Centers for Disease Control and Prevention, the World Health Organization (WHO), and the UK Department of Health declared *K. pneumoniae* infections a serious public health problem.^6^ In most cases,*K. pneumoniae* infections are resistant to clinically used antibiotics. Differences in the severity of infections may have an association with the presence or absence of virulence factors and antibiotic resistance in bacteria.^7^

*K. pneumoniae* consists of different virulence genes such as *kfu* (codes for an iron uptake system^8^), *ybtS*, *mrkD*, *entB* and *iutA*, *rmpA* (regulator of mucoid phenotype A), *allS* (associated with allantoin metabolism), *fimH*, *wabG* (endotoxin‐related genes), *ureA*, *pagO* (transporter gene), aerobactin, and capsule‐associated genes including *wcaG* and *magA*.^9^, ^10^, ^11^ Moreover, wcaG is an endotoxin‐related gene.^12^ The ybt locus comprising ybtS + ybtX + ybtQ + ybtP + ybtA + irp2 + irp1 + ybtU + ybtT + ybtE + fyuA is required for yersiniabactin synthesis.^13^, ^14^ The mrk cluster comprising five genes (mrkABCDF) encodes type 3 fimbriae. mrkD is a subunit of this cluster and is responsible for binding to collagen molecules.^15^, ^16^ *ureA* gene is a subunit of urease and is related to the invasion ability of bacteria.^12^ In general, enterobactin (Ent) encodes a *K. pneumoniae* siderophore. Ent biosynthesis gene (*entB*) with other siderophore encoding genes (*ent*A, C, D, E, or F) and *iutA* have the main role in the pathogenicity of *K. pneumoniae* strains.^17^, ^18^ Type 1 fimbriae are the main adhesive organelles in bacteria belonging to the *Enterobacteriaceae* family. This type of fimbriae is composed of several subunits including FimA, FimF, FimG, and FimH. FimH is an allosteric protein and has a crucial role in attachment.^19^, ^20^

Among virulence factors, the capsule is very important during infection because it confers a mucoid phenotype. The capsule protects the bacterium from phagocytosis by macrophages. The capsule of *K. pneumoniae* has 79 different serotypes. However, serotypes K1 and K2 are quite important and are associated with increased pathogenicity and high mortality rates in many developed and developing countries.^21^, ^22^

As explained so far, the genes encoding virulence‐associated proteins and the capsule of *K. pneumoniae* have main roles in attachment, colonization, invasion, and pathogenicity. The assessment and identification of bacterial virulence factors are useful assets for the design and development of new molecular detection assays and therapeutic pathways. In this regard, the current study aimed to investigate the capsular serotypes, and molecular profiles of genes encoding virulence‐associated factors, in clinical *K. pneumoniae* isolates.

## 2 MATERIALS AND METHODS

### 2.1 Bacterial isolates and study subjects

This cross‐sectional study was carried out on the 950 different clinical samples collected from four teaching hospitals including Imam Khomeini, Buali, Alavi, and Fatemi in Ardabil province, Iran from October 1, 2019, to November 31, 2021. The frequency of different clinical samples was as follows: blood (*n* = 284; 29.9%), urine (*n* = 457; 48.1%), tracheal aspiration (*n* = 123; 12.9%), and catheter (*n* = 86; 9%).

The diagnosis of *K. pneumoniae* isolates was made based on the following criteria: (i) culture on the microbial culture media namely MacConkey agar, Eosin Methylene Blue (EMB), and blood agar plates (Merck Co.), (ii) Gram‐staining method, (iii) IMVIC (Indole, Methyl red, Voges Proskauer, and Citrate) test, (iv) oxidase and catalase test, (v) culture on Triple Sugar Iron Agar, (vi) motility test, (vii) SH2 production, (vii) ONPG (O‐Nitrophenyl‐β‐d‐galactopyranoside) test, (viii) urea test (Sigma‐Aldrich), and (ix) lysine decarboxylase test (HiMedia).

The final identification of *K. pneumoniae* isolates was accomplished using a polymerase chain reaction (PCR) assay and a specific primer pair (SinaClon) including the forward primer: 5′‐TGATTGCATTCGCCACTGG‐3′ and reverse primer: 5′‐ GGTCAACCCAACGATCCTG‐3′ targeted *khe* gene. For this purpose, the total genomic DNA was extracted from *K. pneumoniae* isolates cultivated in LB (Luria–Bertani) liquid microbial growth medium using the DNA extraction kit (AllPrep DNA minikit [Qiagen, Inc.]). The DNA extraction procedure was performed according to the manufacturer's instruction and was stored at −80°C until used for PCR. In addition, a PCR assay was conducted based on the study previously conducted by Jian‐li et al.^10^ Briefly, PCR reactions were performed at a final volume of 25 μL with 12.5 μL of 2 × Master Mix (SinaClon PCR Master Mix) including 1 × PCR buffer, 0.4 mmol/L dNTPs, 3 mmol/L MgCl_2_, and 0.08 IU Taq DNA polymerase, 0.5 μL of 10 pmol of forward primer, 0.5 μL of 10 pmol of reverse primer, 4 μL of extracted DNA, and 7.5 μL of sterile distilled water.

The PCR reaction was conducted using a thermal cycler (Eppendorf, Mastercycler Gradient; Eppendorf, Hamburg, Germany). PCR was performed in 35 cycles including 94°C for 5 min (1 cycle), 94°C for 45 s, annealing at 55°C for 40 s, and 72°C for 1 min. The extension was performed at 72°C for 5 min. Finally, 1.5% agarose gel was prepared and PCR products were screened under UV light. The ladder mix size was 100 bp.

### 2.2 Virulence‐associated gene detection

The presence of virulence‐associated genes including *Kfu*, y*btS*, *mrkD*, *entB*, *iutA*, *rmpA*, *allS*, *fimH*, *wcaG*, *magA* (serotype K1), *pagO*, and aerobactin (*iroD*) was confirmed by PCR. Table 1 lists the sequences of all primers used for detecting the virulence‐associated genes and the length of expected PCR products. PCR was carried out on a thermal cycler (Eppendorf, Mastercycler Gradient; Eppendorf) at the final volume of 25 μL mixture containing 12 μL of x2 PCR Master Mix including 2.5 μL buffer TBE X10, 0.8 μL Mgcl2 (50 mM), 0.5 μL dNTPs (10 mM), 0.3 μL Taq DNA polymerase (5 unit), 1 μL forward primer (80 pmol), 1 μL reverse primer (80 pmol), 3 μL template DNA, and sterile distilled water up to 25 μL. The PCR Master Mix was purchased from Pishgam Company (Amplicon, Pishgam; Cat. no. PR901635). The used PCR conditions were based on the previously published study performed by Jian‐li et al.^10^ All PCR products were stained by DNA‐safe stain (SinaClon Co.) and screened on a 1.5% agarose gel under UV light.

**Table 1:** The sequences of primers used for the detection of virulence‐associated genes and capsular serotypes of *Klebsiella pneumoniae* isolates.

Genes		Sequence (5′ → 3′)	PCR product size (bp)	References
*Kfu*	F	GGCCTTTGTCCAGAGCTACG	638	[10]
	R	GGGTCTGGCGCAGAGTATGC		
y*btS*	F	GACGGAAACAGCACGGTAAA	242	[10]
	R	GAGCATAATAAGGCGAAAGA		
*mrkD*	F	AAGCTATCGCTGTACTTCCGGCA	340	[10]
	R	GCGTTGGCGCTCAGATAGG		
*entB*	F	GTCAACTGGGCCTTTGAGCCGTC	400	[10]
	R	TATGGGCGTAAACGCCGGTGAT		
*iutA*	F	GGGAAAGGCTTCTCTGCCAT	920	[10]
	R	TTATTCGCCACCACGCTCTT		
*rmpA*	F	CATAAGAGTATTGGTTGACAG	461	[10]
	R	CTTGCATGAGCCATCTTTCA		
*allS*	F	CATTACGCACCTTTGTCAGC	764	[10]
	R	GAATGTGTCGGCGATCAGCTT		
*fimH*	F	GCCAACGTCTACGTTAACCTG	180	[10]
	R	ATATTTCACGGTGCCTGAAAA		
*wcaG*	F	GGTTGGKTCAGCAATCGTA	169	[10]
	R	ACTATTCCGCCAACTTTTGC		
*magA (K1)*	F	GGTGCTCTTTACATCATTGC	1283	[10]
	R	GCAATGGCCATTTGCGTTAG		
*pagO*	F	TGCTCTTGAAACTATCCCTCC	244	[10]
	R	GGCAATAACTCCCGTCCA		
*iroD*	F	GCATAGGCGGATACGAACAT	556	[10]
	R	CACAGGGCAATTGCTTACCT		
K2	F	GACCCGATATTCATACTTGACAGAG	641	[10]
	R	CCTGAAGTAAAATCGTAAATAGATGGC		
K5	F	TGGTAGTGATGCTCGCGA	280	[10]
	R	CCTGAACCCACCCCAATC		
K20	F	CGGTGCTACAGTGCATCATT	741	[10]
	R	GTTATACGATGCTCAGTCGC		
K54	F	CATTAGCTCAGTGGTTGGCT	881	[10]
	R	GCTTGACAAACACCATAGCAG		

### 2.3 Serotyping

The *K. pneumoniae* isolates were serotyped for serotypes K2, K5, K20, and K54 using the PCR method based on the specific primers. Table 1 shows the primer sequences used for serotyping. The volume of the utilized materials and PCR conditions were set based on a study previously conducted by Hasani et al.^22^ Briefly, PCR was carried out as a 25 μL reaction mixture including 1 μL of forward primer (10 pmol), 1 μL of reverse primer (10 pmol), 3 μL of extracted DNA, and 8 μL sterile distilled water added to the 12 μL Master PCR mixture (SinaClon x2 PCR Master Mix). PCR amplification was performed using a thermal cycler (Eppendorf, Mastercycler Gradient; Eppendorf) under the following condition: 95°C for 5 min (1 cycle), followed by 35 cycles of 95°C for 45 s, annealing at 55−60°C (based on the primers for each gene) for 1 min, and 72°C for 45 s. The final extension was performed at 72°C for 5 min. All PCR products were screened on 1.5% agarose gel.

### 2.4 Data analysis

All clinical data and results of all tests were included in an SPSS file Version 23 (SPSS Inc.) and the frequency distribution of all data was calculated.

## 3 RESULTS

In total, 100 *K. pneumoniae* isolates were isolated from different clinical samples including blood, urine, tracheal aspiration, and catheter. The highest number of *K. pneumoniae* isolates (*n* = 78/100; 78%) was detected in urine samples. The frequency of *K. pneumoniae* isolates among other clinical samples was as follows: blood (*n* = 12/100; 12%), tracheal aspiration (*n* = 6/100; 6%), and catheter (*n* = 4/100; 4%).

Among all virulence‐associated genes, *entB* (94%), *fimH* (91%), and *wcaG* (87%) exhibited the highest frequency in *K. pneumoniae* isolates (Table 2). All *K. pneumoniae* isolates were negative for *allS*, *pagO*, *magA*, and aerobactin genes.

**Table 2:** Frequency of virulence‐associated genes among 100 *K. pneumoniae* isolates.

Genes	*kfu*	*ybtS*	*mrkD*	*entB*
Frequency (%)	20	39	69	94
Genes	*iutA*	*rmpA*	*allS*	*fimH*
Frequency (%)	4	2	0	91
Genes	*wcaG*	*magA*	*pagO*	Aerobactin
Frequency (%)	87	0	0	0

Of all 100 *K. pneumoniae* isolates, 4% (*n* = 4/100) and 2% (*n* = 2/100) were found to be typeable with K5 and K2 primers, respectively (Table 3). The obtained results indicated that 94% of isolates (*n* = 94/100) were related to non‐K1, K20, and K54 serotypes.

**Table 3:** Frequency of capsular serotypes among 100 *K. pneumoniae* isolates.

Serotypes	K1	K2	K5	K20	K54
Frequency (%)	0	2	4	0	0

Table 4 proves the Coexistence of virulence‐associated genes. In addition, analyses in this study revealed that 24% of *K. pneumoniae* isolates harbored the *entB‐wcaG‐fimH* genes simultaneously. Moreover, 20% of *K. pneumoniae* isolates harbored the *mrkD‐entB‐wcaG‐fimH* genes simultaneously. Results also showed that 50% (*n* = 2/4) of the capsular serotype 5 of *K. pneumoniae* isolates harbored the *ybtS‐mrkD‐entB‐wcaG‐fimH* genes simultaneously.

**Table 4:** Coexistence of virulence associated genes among 100 *K. pneumoniae* isolates.

No.	Virulence‐associated genes	*K. pneumoniae* (*n* = 100)	No.	Virulence‐associated genes	*K. pneumoniae* (*n* = 100)
		*N* (%)			*N* (%)
1	*kfu‐ybts‐mrkD‐entB‐iutA‐wcaG‐fimH*	1/100 (1%)	16	*mrkD‐entB‐wcaG‐fimH*	20/100 (20%)
2^a^	K2*‐ybts‐mrkD‐entB‐wcaG‐fimH*	1/100 (1%)	17	*kfu‐ybts‐mrkD‐entB*	1/100 (1%)
3^b^	K5*‐kfu‐ybts‐mrkD‐entB‐fimH*	1/100 (1%)	18	*kfu‐mrkD‐entB‐fimH*	1/100 (1%)
4^b^	K5*‐ybts‐mrkD‐entB‐wcaG‐fimH*	2/100 (2%)	19^b^	K5*‐entB‐wcaG‐fimH*	1/100 (1%)
5	*kfu‐ybts‐mrkD‐entB‐wcaG‐fimH*	6/100 (6%)	20	*entB‐iutA‐wcaG‐fimH*	1/100 (1%)
6	*ybts‐mrkD‐entB‐iutA‐wcaG‐fimH*	2/100 (2%)	21	*ybts‐mrkD‐entB*	2/100 (2%)
7	*ybts‐mrkD‐entB‐rmpA‐wcaG‐fimH*	1/100 (1%)	22	*ybts‐entB‐wcaG*	1/100 (1%)
8	*ybts‐mrkD‐entB‐wcaG‐fimH*	14/100 (14%)	23	*mrkD‐wcaG‐fimH*	3/100 (3%)
9	*kfu‐ybts‐mrkD‐wcaG‐fimH*	1/100 (1%)	24	*mrkD‐entB‐fimH*	1/100 (1%)
10	*kfu‐ybts‐mrkD‐entB‐fimH*	3/100 (3%)	25	*entB‐wcaG‐fimH*	24/100 (24%)
11	*kfu‐mrkD‐entB‐wcaG‐fimH*	4/100 (4%)	26	*mrkD‐entB*	1/100 (1%)
12	*kfu‐mrkD‐entB‐rmpA‐fimH*	1/100 (1%)	27	*entB‐fimH*	1/100 (1%)
13^a^	K2*‐mrkD‐entB‐wcaG‐fimH*	1/100 (1%)	28	*wcaG*	1/100 (1%)
14	*ybts‐mrkD‐entB‐wcaG*	2/100 (2%)	29	*kfu*	1/100 (1%)
15	*ybts‐entB‐wcaG‐fimH*	1/100 (1%)	30	*‐*	‐

^a^ Profile of virulence‐associated genes in capsular serotype 2.

^b^ Profile of variance‐associated genes in capsular serotype 5.

## 4 DISCUSSION

The present study aimed to investigate the capsular serotypes, and molecular profiles of genes encoding virulence‐associated factors, in clinical *K. pneumoniae* isolates*. K. pneumoniae* as a frequent nosocomial pathogen causes different types of infections such as respiratory, urinary, and blood infections that are related to increased morbidity and mortality.^11^ The presence of different virulence factors and capsular serotypes plays a key role in the invasion and virulence of*K. pneumoniae* such as enhancing bacterial resistance to phagocytosis, antimicrobial peptides, and so forth.^17^ According to the obtained results, out of 100 *K. pneumoniae* isolates, 4% and 2% belonged to the capsular serotypes 5 and 2, respectively. Several studies have analyzed the frequency of capsular serotypes of *K. pneumoniae* isolates worldwide. In 2020, Hasani et al. from Iran investigated the serotypes of *K. pneumoniae* isolated from clinical specimens. They stated that among 61 *K. pneumoniae* isolates, capsular serotypes 5 (8.1%), 20 (21.3%), and 54 (29.5%) were the most common serotypes among which serotype 54 was the predominant.^22^ The results obtained by Rastegar et al. (2019; Iran) revealed that serotypes K1 and K2 were the only detected serotypes among 22 hypermucoviscous/hypervirulent*K. pneumoniae* isolates.^21^ In 2021, Liao et al. from China determined the capsular serotypes of*K. pneumoniae* isolated from patients with bacteremia. According to their study, 18.2%, 16.4%, 6.7%, and 3.6% of 54 *K. pneumoniae* isolates were identified as K1, K2, K57, and k54 serotypes, respectively.^23^ In 2007, Yeh et al. conducted a study in Taiwan and stated that among capsular serotypes of *K. pneumoniae* isolates, K1 (34/73; 46.6%) and K2 (15/73; 20.5%) were the most common ones.^24^ In another study performed by Jian‐li et al. from China (2017), 93.3% (*n* = 14/15) of *K. pneumoniae* isolates belonged to serotype K2.^10^ In 2017, Akbari et al. from Iran confirmed that among 65 *K. pneumoniae* isolates, K1 and K2 serotypes with 10.7% and 6.15% frequency were the predominant ones among the serotypes.^10^

As observed, in general, capsular serotypes K1, K2, K4, and K5 are highly virulent and predominant in human infection.^9^, ^25^ The variations in the prevalence and distribution of capsular serotypes among different studies may result from several factors namely the differences in the type of samples, sample size, and study group as well as the diversity in applied detecting methods.^22^, ^26^

In the present study, *entB*, *fimH*, and *wcaG* had the highest frequency in *K. pneumoniae* isolates, respectively. Moreover, according to the analyses in this study, 50% of capsular serotype 5 *K. pneumoniae* isolates harbored the *ybts‐mrkD‐entB‐wcaG‐fimH* genes simultaneously. Aissani et al. investigated the virulence profiles of *K. pneumoniae* strains isolated from different clinical specimens. They found that *fimH‐1*, *mrkD*, *ycfM*, and *entB* genes were the most common virulence genes.^27^ Zhang et al. analyzed the frequency of virulence‐associated genes in*K. pneumoniae* isolates induced by a pyogenic liver abscess in southeastern China. They demonstrated that *mrkD*, *fimH*, and *rmpA* had the highest frequency among the other genes under study.^28^ KUS et al. confirmed that*entB*, *ycfM*, and *mrkD genes* had the highest prevalence among virulence‐associated genes in *K. pneumoniae* isolates.^29^ In a study carried out by Jian‐li et al., *wabG* and *ureA* genes were isolated from 100% of *K. pneumoniae* isolates.^10^ Hasani et al. found that*uge*, *ycfM*, and *wabG* genes were the most common capsule‐associated virulence genes in *K. pneumoniae* isolates.^22^ Among the limitations of this study, it can be mentioned that due to a lack of funds, we were unable to investigate other virulence factors.

## 5 CONCLUSION

From out of 100 *K. pneumoniae* isolates, 6% were typeable and distributed in the two serotypes K5 and K2. Moreover, a majority of *K. pneumoniae* isolates were positive for multiple types of virulence genes. Detection and identification of bacterial virulence‐associated genes factors are useful assets for the design and development of new molecular detection assays and therapeutic pathways.

## AUTHOR CONTRIBUTIONS

**Neda Same Maleki**: Conceptualization; formal analysis; methodology; project administration; writing—original draft; writing—review and editing. **Forough Babazadeh**: Data curation; methodology; writing—original draft. **Mohsen Arzanlou**: Data curation; investigation; methodology; writing—original draft. **Roghayeh Teimourpour**: Conceptualization; investigation; methodology; project administration; supervision. **Hadi Peeri Dogaheh**: Conceptualization; project administration; supervision; writing—original draft.

## CONFLICT OF INTEREST STATEMENT

The authors declare no conflict of interest.

## ETHICS STATEMENT

All experiments were conducted following the Institutional Ethics Committee of the Ardabil University of Medical Sciences (IR. ARUMS.REC.1399.135). The goals of the research were explained to all patients, and written informed consent was signed by all patients.

## TRANSPARENCY STATEMENT

The lead author Roghayeh Teimourpour, Hadi Peeri Dogaheh affirms that this manuscript is an honest, accurate, and transparent account of the study being reported; that no important aspects of the study have been omitted; and that any discrepancies from the study as planned (and, if relevant, registered) have been explained.

## Data Availability

All data generated or analyzed during this study are included in this published article.
